# Systematic review of cost-effectiveness analyses of weight loss interventions for knee osteoarthritis

**DOI:** 10.1136/bmjopen-2026-117500

**Published:** 2026-09-22

**Authors:** Yue Gao, Hema Mistry, Fatema Dhaif, Blair Mcmurray, Sanjana Susarla, Seyran Naghdi

**Affiliations:** 1 University Hospitals Coventry and Warwickshire NHS Trust, Coventry, UK; 2 University of Warwick, Warwick Medical School, Coventry, UK; 3 George Eliot Hospital NHS Trust, Nuneaton, UK

**Keywords:** Bariatric Surgery, Obesity, Systematic Review

## Abstract

**Abstract:**

**Objectives:**

We evaluated the cost-effectiveness of weight-loss interventions for adults with knee osteoarthritis (OA) and obesity.

**Design:**

Systematic review and narrative synthesis.

**Data sources:**

MEDLINE, EMBASE via Ovid, Global Index Medicus and Web of Science and a clinical trial registry (ClinicalTrials.gov), published up to June 2025.

**Eligibility criteria:**

English-language studies evaluating the cost-effectiveness of intentional weight-loss interventions in adults (≥18 years) with knee OA were included. Studies without cost-effectiveness outcomes, abstracts and conference proceedings were excluded.

**Data extraction and synthesis:**

Two reviewers independently extracted study characteristics and economic outcomes. A narrative synthesis was conducted and costs were standardised to 2024 British pounds.

**Results:**

1014 references were identified; eight studies met inclusion criteria, including six model-based economic evaluations and two randomised trials. Most studies used lifetime horizons and presented data from healthcare or societal perspectives. Combined diet and exercise programmes showed quality-adjusted life years (QALY) gains of 0.05–0.16 with incremental cost-effectiveness ratios (ICERs) of £16 915 and £31 305 per QALY, while bariatric surgery yielded larger gains (0.81–1.7 QALYs) at lower ICERs (£4361 to £19 303 per QALY). Pharmacological interventions were effective but costly with ICER (£32 265 to £35 832 per QALY). Evidence regarding telehealth-only interventions was mixed, with reported ICERs ranging from no significant effect to £32 722 per QALY gained. The included studies fulfilled 79–86% of the criteria from Consolidated Health Economic Evaluation Reporting Standards (CHEERS) checklist and were all rated good on the Drummond checklist.

**Conclusions:**

Using standard UK cost-effectiveness thresholds (£25 000 to £35 000 per QALY), bariatric surgery appears most likely to be cost-effective for patients with knee OA, although this conclusion is based primarily on US model-based evidence. Pharmacological therapies may be cost-effective, although their economic value is sensitive to drug pricing and willingness-to-pay thresholds. High-quality UK-based economic evaluations are needed to establish the cost-effectiveness of Glucagon-Like Peptide-1 (GLP-1) receptor agonists for knee OA.

**STRENGTHS AND LIMITATIONS OF THIS STUDY**

- This systematic review followed Preferred Reporting Items for Systematic Reviews and Meta-Analyses (PRISMA) guidance, with a prospectively registered protocol and included both trial-based and model-based economic evaluations.
- Costs and cost-effectiveness outcomes were standardised to a common currency and price year to improve cross-study comparability.
- Most included studies were model-based and relied on assumptions regarding long-term costs and outcomes.
- Considerable methodological heterogeneity across interventions and study designs limited direct comparability.

## Introduction

 The global disease burden of chronic, disabling conditions such as knee osteoarthritis (OA) and obesity is increasing. Projections show that by 2050, cases of knee OA will increase by 74.9%,^1^ and nearly 60% of the entire world population is expected to be overweight or obese.^2^ In the UK, the annual cost of obesity has been estimated at £126 billion and is projected to reach £150 billion by 2035.^3^ Estimates from York Health Economics Consortium indicate that the combined healthcare costs of OA and rheumatoid arthritis over the next decade could total £118.6 billion.^4^ Notably, musculoskeletal conditions such as knee OA represent the highest annual cost per case associated with obesity, at £27 798.40 per patient.^5^

Obesity is a well-established, modifiable risk factor for both the onset and progression of knee OA, contributing to joint degeneration, pain and functional limitation.^6^ Individuals affected by both obesity and knee OA often experience a reduced quality of life and increased healthcare usage.^7^ Addressing knee OA symptoms only after they emerge is often insufficient to prevent excessive healthcare utilisation, and may result in prolonged decline in quality of life while patients await effective pain management or surgical interventions.^8^ Efforts should instead focus on addressing the underlying contributors, such as obesity. Achieving effective weight reduction can slow disease progression, enhance clinical outcomes and ultimately reduce long-term treatment costs.^9^ However, it remains unclear which weight-loss strategies provide the best value for money in people with knee OA.

Previous systematic reviews have highlighted the benefits of diet and exercise interventions but have been limited in their exploration of the long-term economic advantages of weight loss strategies.^10 11^ In addition, the cost-effectiveness of newer pharmacological treatments and contemporary surgical pathways has not been comprehensively assessed, and no UK-specific economic evaluation currently exists. This review aims to evaluate the cost-effectiveness of surgical and pharmacological interventions alongside established diet and exercise approaches, to inform healthcare decision-making and future policy development.

## Methods

We conducted a systematic review and reported our findings in accordance with the Preferred Reporting Items for Systematic Reviews and Meta-Analyses (PRISMA) guidelines and recommendations for systematic review of economic evaluations.^12^ The protocol was registered prospectively on PROSPERO (registration number CRD420251075205).

### Search strategy

The literature search was conducted in line with guidance from the Cochrane handbook.^13^ The search strategy was developed collaboratively with input from an information specialist, clinical experts and health economists. MEDLINE All (Ovid), Embase (Ovid), Science Citation Index (Web of Science), Global Index Medicus (WHO) and ClinicalTrials.gov were searched from inception to June 2025. The search combined controlled vocabulary terms and free-text terms relating to three core concepts: knee OA, obesity, weight management interventions and economic evaluation. Weight management terms covered diet, calorie restriction, behavioural interventions, pharmacological weight loss treatments and bariatric procedures. Economic terms covered cost-effectiveness, cost-utility, economic evaluation, economic models and health utility measures. The full search strategies for each database are provided in online supplemental appendix 1.

### Eligibility criteria

The review included studies evaluating intentional weight-loss interventions in adults (≥18 years) with knee OA. Eligible interventions comprised dietary modification, exercise programmes targeting weight loss, combined diet and exercise interventions, behavioural therapies (eg, cognitive behavioural therapy), digital or remote support (eg, apps or calls), Glucagon-Like Peptide-1 (GLP-1) receptor agonist drugs (including liraglutide, semaglutide and tirzepatide) and bariatric surgery when performed for weight reduction in patients with OA or prior to joint replacement. Any combination of these interventions was also eligible. Comparators included usual care, placebo or no treatment.

We included both randomised and non-randomised study designs, encompassing full economic evaluations (cost-minimisation, cost-effectiveness, cost-consequence, cost-benefit and cost-utility analyses), modelling studies and randomised controlled trials (RCTs) incorporating economic evaluations. There were no restrictions on geographical setting, healthcare system or economic perspective (eg, healthcare provider, societal). Only studies published in English were included. Abstracts, conference proceedings and studies lacking cost-effectiveness outcomes were excluded. Relevant systematic reviews were screened to identify eligible primary studies, which were subsequently included.

### Study selection

All retrieved records were imported into Rayyan.^14^ Two reviewers (YG and BM) screened titles and abstracts using the eligibility criteria. Disagreements were resolved through discussion and, where necessary, consultation with a third reviewer (SS). Full texts of suitable abstracts were screened by two independent reviewers (YG and BM). Where full texts were unavailable, corresponding authors were contacted.

### Data extraction

Data from included studies were then independently extracted (YG and SS) into a Microsoft Excel file. Any conflicts were resolved in consensus meeting with input from the third reviewer (BM). Extracted data were predetermined using the Consolidated Health Economic Evaluation Reporting Standards (CHEERS) checklist.^15^ These included study characteristics (author, year, type of study, type of economic evaluation, model type, country, population, intervention, control, main outcomes measured, discount rate, time horizon) and results (perspective, weight loss intervention, direct intervention cost, quality-adjusted life year (QALY), discount rate, incremental cost-effectiveness results (ICER), probability analyses, cost-effectiveness threshold and results).

### Synthesis methods

A narrative synthesis was conducted. To enhance comparability across studies, all reported costs were standardised to 2024 British pound sterling (GBP). Cost data were first inflated to 2024 values in the original local currency using country-specific Gross Domestic Product (GDP) deflator indices and subsequently converted to GBP using purchasing power parity (PPP) exchange rates for GDP. Conversions were performed using the Campbell and Cochrane Economics Methods Group (CCEMG) and Evidence for Policy and Practice Information and Co-ordinating Centre (EPPI) cost converter. The converter draws on GDP-based PPPs and GDP deflators from the International Monetary Fund World Economic Outlook database.^16^

ICERs were recalculated using the following formula to ensure consistency across studies (full data tables available in online supplemental appendix 2):


$$ICER_{\left\{New\right\}}=\frac{\left(ICER_{\left\{Intervention,study\right\}}-ICER_{\left\{Comparator,study\right\}}\right)}{EffectSize_{\left\{study\right\}}}$$

Where:

- ICER_{New}_ is the incremental cost-effectiveness ratio used in the systematic review.
- ICER_{Intervention, study}_ is the ICER for the intervention arm converted from the original study’s ICER using the CCEMG–EPPI cost converter to reflect the price year and currency of the current study.
- ICER_{Comparator, study}_ is the incremental cost-effectiveness ratio for the comparator arm converted from the original study’s ICER using the CCEMG-EPPI cost converter to reflect the price year and currency of the current study.
- Effect Size_{study}_ is the original incremental effectiveness, measured as the difference in QALYs between the intervention and comparator in the original study.

### Cost-effectiveness thresholds

In the UK, the National Institute for Health and Care Excellence (NICE) recommends that interventions with an ICER between £25 000 and £35 000 per QALY are considered cost-effective.^17^ The majority of existing studies apply the US willingness-to-pay (WTP) threshold of US$100 000 per QALY. To reflect the studies as reported while enabling cross-study comparability, we therefore present results using their original WTP benchmark, expressed in a common currency and price year. Cost-effectiveness thresholds reported in the original studies were converted to 2024 GBP sterling using the same CCEMG-EPPI cost converter and assumptions applied to ICERs (GDP-based PPP and GDP deflators). Converted thresholds represent the original WTP values and do not imply application of UK threshold, which were considered separately in the interpretation.

### Quality assessment

The methodological quality and reporting quality of included studies were assessed according to study design. Reporting quality was assessed using the CHEERS 2022 checklist^15^; a 28-item reporting guideline designed to evaluate the completeness and transparency of reporting in economic evaluations. Each item was recorded as either reported (‘yes’) or not reported (‘no’). Methodological quality was assessed using Drummond’s checklist for the critical appraisal of economic evaluations,^18^ which evaluates key aspects of study design, data collection, analysis and interpretation. Each checklist item was assessed as fully addressed (‘yes’), partially addressed (‘partial’), not applicable or not addressed (‘no’). Both assessments were conducted independently by two reviewers (YG and SS), with any disagreements resolved through discussion and consensus.

### Patient and public involvement

Patients and the public were not involved in this systematic review.

## Results

**Figure 1:**
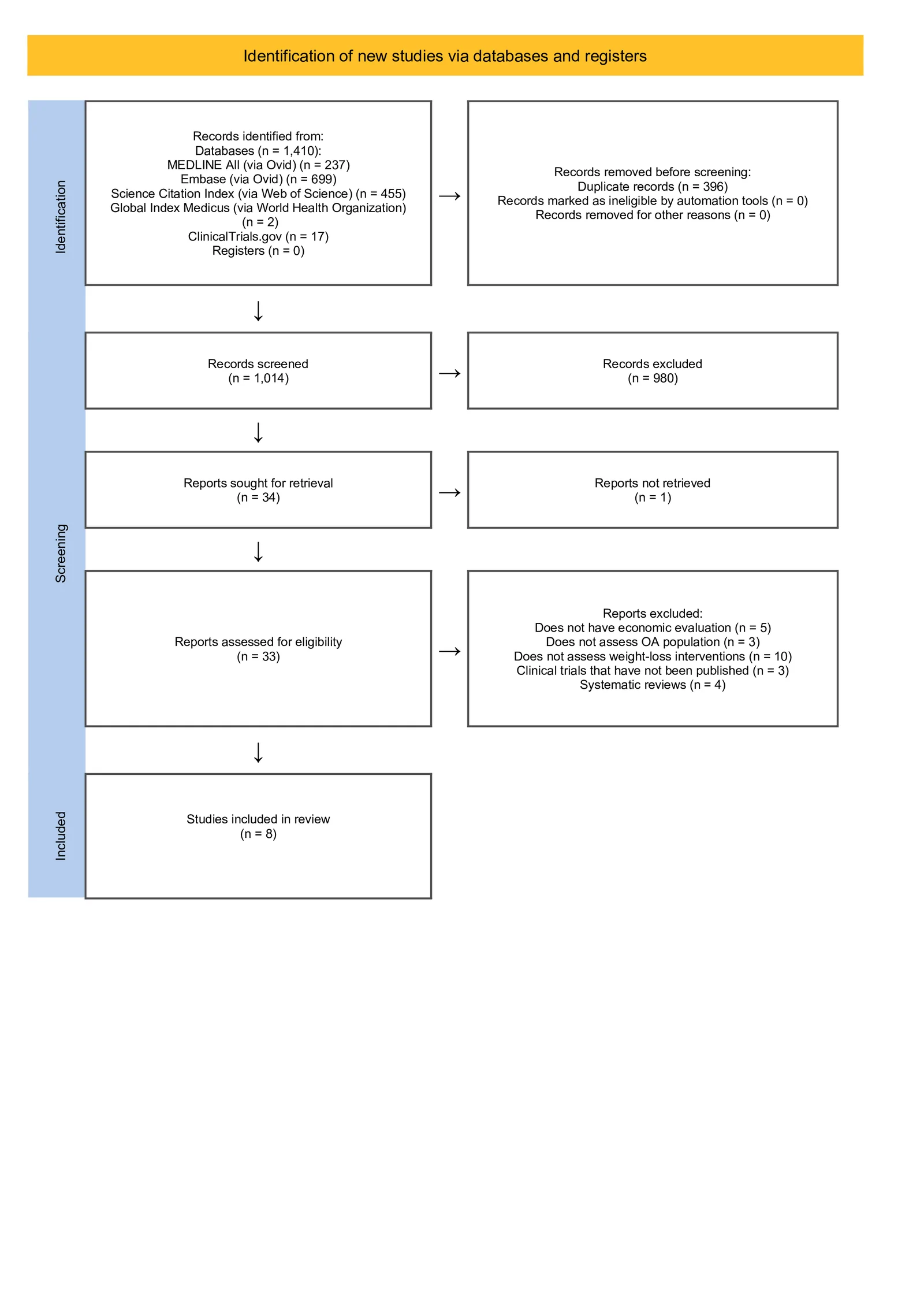
PRISMA flow diagram for study selection.^38^ OA, osteoarthritis; PRISMA, Preferred Reporting Items for Systematic Reviews and Meta-Analyses.

The search strategies yielded 1014 records after the removal of duplicates. Following title and abstract screening, 980 records were excluded. One report could not be retrieved as it lacked a valid Digital Object Identifier (DOI) and was not published under the authors’ stated name. 33 full text articles were reviewed, and 8 studies met the inclusion criteria^19–26^ and were included in this review. Figure 1 online supplemental appendix 3.

### Study characteristics

These eight studies were published between 2016 and 2025 and were conducted in the USA (n=6) and Australia (n=2). All eight studies^19–26^ conducted a cost-utility analysis and one study additionally reported a cost-effectiveness analysis.

Six studies^19–24^ used computer simulation models to evaluate long-term cost-effectiveness. Five of these^19–23^ employed the Osteoarthritis Policy (OAPol) Model, while one study used a Markov model.^24^ All applied a 3% annual discount rate and projected outcomes over lifetime horizons.

Modelled populations were derived from real-world cohort data from previous clinical trials such as the Weight-Loss and Exercise for Communities with Arthritis in North Carolina,^27^ Intensive Diet and Exercise for Arthritis^28^ and Longitudinal Assessment of Bariatric Surgery study,^29^ as well as Semaglutide Treatment Effect in People with obesity for GLP-1 receptor agonist pharmacotherapy.^30^ Detailed cohort characteristics used in each model are detailed below.

Two studies^25 26^ conducted RCTs that collectively assessed 544 participants with knee OA over a 12-month period. These trials derived cost-utility ratios from Western Ontario and McMaster Universities Osteoarthritis Index scores and Assessment of Quality of Life instrument. Online supplemental table 1 summarises the general characteristics of the included studies.

Seven out of the eight studies^19–24 26^ reported their interventions to be cost-effective, with ICER/incremental cost-utility ratio being below the WTP threshold applied in each study. Key economic outcomes are summarised in table 1.

**Table 1:** Key results

Author	Perspective	Weight loss intervention	QALY diff.	Standardised ICER/ICUR to 2024 GBP (rounded to the nearest £)	Method of sensitivity analysis	Probability analysis	Cost-effectiveness threshold used in study	Conclusions from studies (cost-effective compared with control?)
McLawhorn *et al*^24^	Societal	Bariatric surgery	1.7	13 021	One-way and two-way	98.80%	US$100 000 per QALY	Yes
Kopp *et al*^19^	Healthcare	Health education	0.07	13 375	One-way and two-way	N.R	US$100 000 per QALY	Yes
	Societal		0.08	10 664				
	Healthcare	Diet and exercise	0.12	31 305		60%		
	Societal		0.13	27 694				
Losina *et al*^21^	Healthcare	Diet and exercise	0.054	31 150	One-way	58% at US$50 000 100%at US$100 000	US$50 000 to US$100 000 per QALY	Yes
	Societal		0.054	27 871		68% at US$50 000 100% at US$100 000		
Kostic *et al* 2023 March	Healthcare	Roux-en-Y gastric bypass	1.36	4361	One-way and two-way	70%	US$50 000 per QALY	Yes
		Laparoscopic sleeve gastrectomy	1.14	6038		30%		
Harris *et al*^26^	Healthcare	Telehealth exercise	0.03	32 722	Unclear	53%	$A70 000 per QALY	Yes
		Telehealth diet and exercise	0.05	24 581		88%		
O’Brien *et al*^25^	Healthcare	Telehealth advice	0	0*	Unclear	0%	$A100 000 per QALY	No
	Societal		0	0*		0%		
Betensky *et al*^23^	Healthcare	Exercise and diet	0.16	16 916	One-way and two-way	NR	US$100 000 per QALY	Yes
		Semaglutide	0.92	35 832		34%		
		Tirzepatide	1.14	32 265		64%		
		Laparoscopic sleeve gastrectomy	1.01	14 292		17%		
		Roux-en-Y gastric bypass	1.27	15 683		68%		
Kostic *et al* 2023 August	Healthcare	Roux-en-Y gastric bypass (at 47 BMI)	1.12	16 127	One-way and two-way	N.R	US$100 000 per QALY	Yes
		Roux-en-Y gastric bypass (at 42 BMI)	0.99	19 303				
		Laparoscopic sleeve gastrectomy (at 47 BMI)	0.89	16 036				
		Laparoscopic sleeve gastrectomy (at 42 BMI)	0.81	17 229				
		Lifestyle non-surgical weight loss (at 47 BMI)	0.15	92 393				
		Lifestyle non-surgical weight loss (at 42 BMI)	0.18	76 661				

* Unable to calculate due to no QALY difference.

BMI, body mass index; Dominated, ICER not calculated due to another intervention in the same study produced more QALYs with less cost; GBP, British pound; ICER, incremental cost-effectiveness results; ICUR, incremental cost-utility ratio; N.R, not recorded in the study; QALY, quality-adjusted life year; QALY diff, difference between intervention QALY and control QALY.

### Cost

Costs of weight loss interventions varied substantially by intervention type. They are summarised in online supplemental table 2. Bariatric surgery remained the most expensive approach, with Roux-en-Y gastric bypass (RYGB) and laparoscopic sleeve gastrectomy (LSG) typically costing between £16 694 and £22 374, with some studies reporting total costs up to £23 113 to £31 188 when perioperative care and complications are included. Ongoing follow-up costs were not as expensive but recurring, at approximately £208 per year. Across studies, surgery also produced the largest and most sustained health gains, particularly in terms of long-term weight loss, diabetes remission and improvements in health-related quality of life.^20 22 23^

Pharmacological interventions, including GLP-1 receptor agonists such as semaglutide and tirzepatide, represented an intermediate cost category. Reported total costs ranged from approximately £2984 to £7287 depending on drug choice, dosing regimen and formulation. Tirzepatide tended to be at the upper end of this range, reflecting higher acquisition costs but also greater average weight loss and metabolic benefit.^23^

Lifestyle-based interventions, including structured diet and exercise programmes delivered in-person, were generally less expensive but still variable depending on intensity and resource use. Costs ranged from approximately £1151 to £8702 over intervention periods of 6–24 months. Lower-cost programmes typically involved group-based education or minimal supervised activity, whereas higher-cost interventions included intensive dietitian input, exercise supervision, meal replacements and gym access.^19 21 23^

Telehealth-delivered interventions were the least costly overall. Telephone or digitally delivered programmes ranged from approximately £321 for exercise-only interventions to £1298 for combined diet and exercise programmes over 6 months. These interventions relied primarily on remote coaching, limited equipment provision and reduced facility costs, making them the most cost-efficient delivery model in terms of direct expenditure.^25 26^

### Surgical interventions

Four simulated studies^20 22–24^ assessed the cost-effectiveness of surgical weight loss interventions, specifically LSG and RYGB. All four studies assumed a strong association between obesity and end stage knee OA requiring total knee replacement (TKR). Across these studies, bariatric surgery was associated with substantial QALY gains ranging from 0.81 to 1.7, with ICERs ranging from £4361 to £19 303 per QALY.

McLawhorn *et al* presented the largest QALY gain (1.7) among all interventions; however, the exact type of bariatric surgery was not specified.^24^ Betensky *et al* was the only study that investigated the cost-effectiveness of LSG and RYGB against a control population not requiring TKR, with ICERs of £14 292 and £15 683, respectively. Probabilistic analysis favoured RYGB as having a higher likelihood of being cost-effective for surgical interventions (68% compared with 17% for LSG) at US$100 000 threshold.^23^ Sensitivity analyses indicated that the cost-effectiveness of bariatric surgery was driven primarily by lower complication rates, reduced reintervention rates and the sustained stability of body mass index over time.

### Diet and exercise

Three studies^19 21 23^ assessed diet and exercise interventions delivered through structured programmes or health education. These interventions generated modest QALY improvements (0.05–0.16) and ICERs between £16 916 and £31 305 per QALY.

Both Kopp *et al* and Losina *et al* found that diet plus exercise programmes offered good value for money from both healthcare and societal perspectives. Kopp *et al* concluded that implementing group nutrition classes and exercise classes with regular meal replacement shakes could be cost-effective up to a WTP threshold of US$100 000 per QALY. However, providing classes aimed at improving knowledge related to nutrition and healthy lifestyle alone provided smaller benefits (0.07–0.08 QALYs), although at a lower cost.^19^

Losina *et al* and Betensky *et al* were less specific about their intervention descriptions. Losina *et al* showed that combined diet and exercise interventions yielded ICERs of £27 871 to £31 150 per QALY, achieving a 58–68% probability of cost-effectiveness at WTP thresholds of US$50 000 per QALY.^21^ Betensky *et al* showed similar effects with diet and exercise programmes achieving a QALY gain of 0.16 and an ICER of £16 916 per QALY, although bariatric surgery produced larger health gains at lower ICERs.^23^

### Telehealth remote weight-loss interventions

Two RCTs conducted in Australia explored telehealth or remotely delivered diet and exercise programmes.^25 26^ Harris *et al* found that a combined diet and exercise telehealth programme generated small but positive QALY gains (0.05), with an estimated ICER of £24 581 per QALY, corresponding to an 88% probability of cost-effectiveness at a $A70 000 per QALY threshold. The exercise-only programme was less cost-effective (£32 722 per QALY), with a 53% probability of being cost-effective at the same threshold. In contrast, O’Brien *et al* reported that a telephone-based coaching intervention was not cost-effective, producing no QALY gains and ICERs were not calculated due to no clinical effect from both healthcare and societal perspectives.^25^

### Pharmacologic weight-loss therapies

Betensky *et al* reported that semaglutide and tirzepatide were cost-effective for patients with knee OA and obesity, with probabilities of cost-effectiveness of 34% for semaglutide and 64% for tirzepatide. Among the two, tirzepatide was identified as more economically favourable at WTP thresholds above US$20 000 per QALY. However, while these agents achieved significant QALY gains (0.92–1.14), their ICER remained moderately high (£32 265 to £35 832 per QALY).

### Quality assessment

All eight studies^19–26^ were evaluated using the CHEERS checklist. In the modelling studies 79–86% of the CHEERS items were reported, whereas for the RCTs 79–82% of items were reported (online supplemental appendix 3). All 8 studies were rated as good quality using the Drummond checklist (online supplemental appendix 4). The main limitation reducing reporting completeness was the lack of description of how impacts are distributed across individuals and the wider population, limited reporting on approaches to engage the cohort and stakeholders in the design, and any differences made by such engagement. Model-based assessments also failed to report mean and CIs for costs and overall outcomes, whereas RCTs frequently failed to report on uncertainty analysis, distributional assumptions and subgroup analysis.

## Discussion

### Main findings

This review identified eight studies^19–26^ that measured or estimated the cost-effectiveness of weight loss interventions for knee OA. Most of these interventions were associated with improvements in QALYs at a WTP threshold of US$100 000 per QALY. Only the use of telehealth-based health promotion resulted in a small increase in QALYs, and its cost-effectiveness remains uncertain. It is important to note that weight loss interventions confer a broad range of health benefits beyond reductions in knee pain, including improvements in cardiometabolic risk, diabetes outcomes, cardiovascular disease risk and overall mortality^31^; these benefits are not consistently or fully captured within the QALY estimates used across the included studies, and therefore may underestimate the true value of these interventions.

The interpretation of cost-effectiveness is complicated by the fact that none of the included studies were conducted in the UK and therefore did not use NICE-aligned economic assumptions or UK-specific costing parameters.^32^ Applying these stricter thresholds changes the interpretation of which interventions might be considered cost-effective in a UK context.

Bariatric surgery is the only intervention that has consistently been shown across non-UK studies to be the most effective at increasing QALYs with a relatively low cost averaged across a patient’s lifetime.^20 22 24 33^ This finding aligns with Boyers *et al* who also found that bariatric surgery was the most cost-effective and most efficient use of resources compared with behaviour or diet therapy by a significant margin.^33^ Nevertheless, because all surgical economic evaluations were conducted in the USA, generalisability to the National Health Service (NHS) is limited by differences in procedure costs, reimbursement pathways, complication rates and surgical eligibility criteria.

Pharmacological options such as semaglutide and tirzepatide show the next greatest potential for improving health outcomes, although their cost-effectiveness is highly sensitive to drug pricing and payer WTP; even a 25% price increase caused tirzepatide to be dominated by other treatment options.^23^ However, Evans *et al* have found even with higher drug acquisition costs, tirzepatide will still be more cost-effective than alternative treatments, with the additional expense offset by significant reductions in costs related to managing comorbidities that occur as a result of obesity.^34^ The true economic value of using GLP-1 agonists in knee OA therefore remains uncertain and is likely to differ substantially between healthcare systems. Drug acquisition costs are substantially higher in the USA than in the UK. Tirzepatide has been estimated to cost £2984 per year in the USA, whereas in the UK annual costs range from £1596 to £3960 depending on dose, and are generally lower.^23 35^ Further UK-based economic evaluations are required before firm policy recommendations can be made.

Diet and exercise are a well-documented cost-effective measure of weight loss in patients with OA. Mazzei *et al* have also demonstrated that structured diet and exercise programmes are cost-effective compared with usual treatments.^11^ Although diet and exercise interventions yielded smaller QALY gains, their ICERs generally remained within the £25 000 to £35 000 per QALY range used for interpretation. Nevertheless, exercise has been shown to improve weight loss maintenance when used in combination with GLP-1 agonist therapy, but the health economic implications of this have not been adequately explored.^36^ Furthermore, there is substantial heterogeneity in how diet and exercise interventions were defined, delivered and measured across studies and more precise intervention descriptions and standardised outcome reporting would improve comparability.

### Strength and limitations

This review provides evidence on the long-term cost-effectiveness of interventions that were not captured in previous systematic reviews. Comparability of international economic evidence was enhanced by standardising reported ICERs and WTP thresholds to a common currency and price year. In addition, study findings were evaluated against UK cost-effectiveness thresholds to aid interpretation for UK decision-making contexts.

Despite its strengths, this review has some limitations. First, converting costs to GBP does not address deeper structural differences such as healthcare system organisation, salary structures, intervention delivery costs or patient pathways and therefore should not be interpreted as making the estimates directly applicable to the UK NHS. Most of the studies included in this review are model-based projections rather than real-world data. These model-based projections rely heavily on assumptions about disease progression, costs and patient behaviour. Extrapolating from the relatively short time horizons of many included studies—often ranging from 12 to 24 months—may limit the ability to fully capture long-term cost-effectiveness. As a result, potential delayed or sustained effects of interventions, including both benefits and adverse events, may not be fully reflected in the reported ICER estimates.

This is especially problematic in instances where insufficient primary research has taken place to build confidence in the assumptions used in economic models. For example, semaglutide and tirzepatide are diabetes medications which have only been recently approved as weight loss treatment, therefore, assumptions made about the length of treatment required, long-term societal and healthcare costs are largely based on data from the last 4 years.^36^ Robust economic evaluation requires evidence on the durability and long-term sustainability of clinical benefits; however, such data are currently limited and are unlikely to be available until the mid-term to long-term.

Second, different interventions were grouped together as general comparisons even though there is significant heterogeneity between studies. This is evident in comparisons between diet and exercise; the specifics of what diet and exercise measures are not always detailed in the studies. There may be significant in programme intensity, duration and modality which are not always assessed, giving inaccurate economic effects and QALY differences. Similarly, telehealth interventions ranged widely in format, content and clinician involvement, limiting the ability to generalise findings across digital health modalities.

Third, several studies used the OAPol model, which is a validated model that enhances comparability across evaluations. The use of this model may introduce structural dependence, whereby cross-study consistency partly reflects a common model structure rather than independent replication. Therefore, readers should interpret these OAPol-based studies with appropriate caution.

### Implications for policy

The widespread adoption of standardised OAPol models allows for greater comparability across economic evaluations. As a result, consistent findings from multiple studies could form the basis for policy changes. Bariatric surgery has a high probability of being cost-effective at a £25 000 to 35 000 threshold. This effect is long lasting and seems to have moderate to high QALY impact. Therefore, eligible patients with end stage knee OA and obesity should be considered for bariatric surgery prior to a TKR. However, policy recommendations should be cautious, as UK-specific costings and surgical capacity constraints were not incorporated into any included studies.

Diet and exercise appear to be cost-effective and have already been implemented in clinical guidelines across the globe but further policy around treatment adherence may be required to maximise their cost-effectiveness.^37^ Pharmacological weight loss therapy may be the future of obesity management; however, currently there is insufficient legislation to help support the cost issue associated with newer GLP-1 agonists. Further policies may need to evaluate price-negotiation, prescribing restrictions or outcomes-based reimbursement schemes to ensure that long-term cost-effectiveness is preserved.

### Future research

Future research should prioritise RCTs evaluating the long-term effectiveness and economic value of pharmacological weight-loss therapies in knee OA. Key priorities include head-to-head comparisons of GLP-1 agonists, assessment of durability of weight loss and symptom improvement beyond 2 years and trial-embedded cost-utility analyses that reflect UK payer thresholds. Studies should also investigate optimal dosing and maintenance strategies, the impact of these agents on structural joint outcomes and whether specific patient subgroups derive greater benefit. Combination therapy trials integrating pharmacological agents with structured lifestyle interventions may further clarify synergistic effects. Collectively, such evidence is needed to define the appropriate role of pharmacological weight-loss treatments in the management pathway for knee OA.

## Conclusion

This systematic review suggests that weight-loss interventions for people with knee OA and obesity may offer clinical benefit, but the current evidence base is limited by the small number of available studies, variation in intervention types and settings and substantial heterogeneity in model structures, assumptions and time horizons.

Across the included studies, bariatric surgery appeared favourable in long-term economic models, with potential for QALY gains and downstream cost offsets. However, these findings were derived from non-UK studies and depended on modelling assumptions regarding the durability of weight-loss and long-term health benefits. As such, their direct applicability to NHS decision-making remains uncertain.

Pharmacological therapies such as semaglutide and tirzepatide demonstrated potential clinical benefit, but their estimated cost-effectiveness was sensitive to drug acquisition costs, treatment duration, adherence and assumptions about sustained benefit after discontinuation. Lifestyle interventions were generally associated with smaller health gains but may remain important components of care given their potential role alongside other weight-loss strategies.

Overall, the available evidence indicates that weight-management interventions may have a role in improving outcomes for people with knee OA and obesity, but there is insufficient high-quality evidence to determine the optimal strategy or sequence of interventions. Further UK-based economic evaluations ideally conducted alongside high-quality randomised trials with longer-term follow-up are needed to inform policy and guide resource allocation in this population.

## Supplementary material

10.1136/bmjopen-2026-117500 online supplemental file 1

10.1136/bmjopen-2026-117500 online supplemental file 2

## Data Availability

Data are available upon reasonable request.
