# Supplementary material for: Systematic review of cost-effectiveness analyses of weight loss interventions for knee osteoarthritis
Source: BMJ Open. 2026 Sep 22;16(9):e117500. doi: 10.1136/bmjopen-2026-117500 (PMC13599885; doi:10.1136/bmjopen-2026-117500)
Supplement: Supplementary file 1 [file bmjopen-2026-117500.reviewer_comments.pdf]

PEER REVIEW HISTORY

BMJ Open publishes all reviews undertaken for accepted manuscripts. Reviewers are asked to complete a checklist review form and are provided with free text boxes to elaborate on their assessment. These free text comments are reproduced below.

ARTICLE DETAILS

Title (Provisional)

Systematic review of cost-effectiveness analyses of weight loss interventions for knee osteoarthritis

Authors

Gao, Yue; Mistry, Hema; Dhaif, Fatema; Mcmurray, Blair; Susarla, Sanjana; Naghdi, Seyran

---

VERSION 1 - REVIEW

**Reviewer**	**1**
**Name**	**Lawford, Belinda**
**Affiliation**	**The University of Melbourne**
**Date**	**16-Mar-2026**
**COI**	**None**

Thank you for the invitation to review this systematic review of economic evaluations examining the cost-effectiveness of weight loss interventions for knee OA. I have a couple of comments that will hopefully help strengthen the manuscript:

1. There appears to be a bit of a mismatch between the results and the conclusions – particularly in the abstract. For example, the abstract states that telehealth-only interventions are not cost-effective. Yet in the results, the description of one of the telehealth-delivered diet and exercise interventions is within the £20-£30K per QALY threshold. The wording should reflect this nuance.

2. Only 8 studies were included in the review, and they appeared to vary substantially in terms of population characteristics, analyses (e.g., time horizon), interventions (e.g., within the 'diet and exercise' group there is substantial variation in type of diet etc) and the fact that only two studies were embedded within an RCT. Further, most studies were conducted in different countries and healthcare contexts. As such, I think the conclusions (and language used in the discussion in places) need to be tempered, warning readers that findings are uncertain given the limited evidence and heterogeneity.

3. The CHEERs checklist was used to assess reporting quality. However, this is a reporting guideline rather than a methodological quality tool. Would something like 'Drummond's checklist for the critical appraisal of economic evaluations' be more appropriate?

4. In Table 1, some of the intervention descriptions are very vague – particularly around 'diet and exercise' interventions. There are a multitude of diets and they vary substantially in costs and intensity. Could any more information be added? E.g., Were they very low energy diets involving meal replacements and dietitian consultations, or were they general healthy eating advice from a GP? These would vary significantly in effectiveness as well as direct healthcare costs.

Reviewer 2

Name Herrmann, Stephan

Affiliation University of Freiburg Medical Center Freiburg, Department of General and Visceral Surgery

Date 26-May-2026

COI None

Thank you very much for the opportunity to review this manuscript.

The study objective is clearly defined, the methodology is appropriate, and the overall structure of the manuscript is well organized. A major strength of this systematic review is the use of standardized ICERs together with a clearly defined NICE-appropriate WTP threshold, making the findings particularly relevant to the UK healthcare setting.

However, several aspects would benefit from further clarification and refinement:

1) Please report an explicit assessment of the methodological quality of included economic evaluations, as well as an appraisal of model validity. CHEERS alone is insufficient, as it assesses reporting quality rather than methodological rigor or validation.

2) The repeated use of the OAPol model in 5/8 included studies may introduce structural dependence across studies, thereby limiting the independence of the evidence base. This is particularly relevant with respect to the transferability across different healthcare systems, populations, and reimbursement contexts, such as those relevant to the UK. This should be addressed in more detail.

3) You should consider addressing the relatively short observation period of oftentimes 12 to 24 months in many studies. This may limit the ability to fully capture long-term cost-effectiveness, particularly with respect to disease progression in knee osteoarthritis as well as TKR and associated downstream costs (e.g. complications). Furthermore, potential delayed or sustained effects (including adverse events) of the evaluated interventions could

be not fully captured. In addition, the uncertainty and robustness of the reported ICER estimates should be explicitly discussed.

4) For emerging pharmacological therapies, evidence on the durability and long-term sustainability of clinical benefits will be essential for robust economic evaluation. However, such data are currently limited and are likely to become available only in the mid- to long-term. This should also be acknowledged

---

VERSION 1 - AUTHOR RESPONSE

Reviewer 1 (Dr Belinda Lawford)

Comment 1

There appears to be a mismatch between the results and conclusions, particularly regarding telehealth interventions.

Response

We revised the Abstract, Results, Discussion, and Conclusions sections to reflect the heterogeneity of findings among telehealth interventions. The revised text acknowledges that while some telehealth-only interventions exceeded conventional willingness-to-pay thresholds, at least one telehealth-delivered diet and exercise intervention demonstrated ICERs within the NICE threshold range. Accordingly, our conclusions now reflect greater nuance and avoid broad statements regarding telehealth interventions as a whole.

Comment 2

The conclusions should be tempered given the small number of studies and substantial heterogeneity.

Response

We agree that the conclusions should be more cautious given the small number of studies and substantial heterogeneity. We have revised the conclusion to temper the interpretation of findings and avoid making definitive recommendations about the optimal weight loss strategy and emphasise uncertainty and need for further UK based economic evaluations.

Comment 3

CHEERS is a reporting guideline rather than a methodological quality tool.

Response

In addition to CHEERS, we conducted a methodological quality assessment using Drummond's Checklist for Economic Evaluations (or alternative tool used). The

rationale, assessment procedures, and findings have been added to the Methods and Results sections.

Comment 4

Intervention descriptions in Table 1 are vague.

Response

Table 1 has been expanded to include additional information regarding intervention characteristics, including dietary approach, exercise components, delivery mode, intensity, duration, healthcare professionals involved, and where applicable, meal replacement or behavioural support components.

Reviewer 2 (Dr Stephan Herrmann)

Comment 1

Please report an explicit assessment of methodological quality and model validity.

Response

We incorporated a formal methodological quality assessment using Drummond's Checklist and added a narrative appraisal of model validity. The revised manuscript now discusses model structure, assumptions, calibration, validation procedures where reported, and implications for interpretation of findings.

Comment 2

The repeated use of the OAPol model may introduce structural dependence across studies.

Response

We thank the reviewer for highlighting the issue of structural dependence associated with repeated use of the OAPol Model. We agree that reliance on the same model can cause correlated structural dependence. However, the OAPol is a validated model that enhances comparability across evaluations. We have revised the manuscript to explicitly acknowledge this consideration and to ensure that this is interpreted with caution.

Comment 3

Short observation periods may limit long-term cost-effectiveness assessment.

Response

We expanded the Limitations section to discuss how relatively short follow-up periods (typically 12–24 months) may not fully capture long-term disease progression, total

knee replacement rates, downstream healthcare costs, complications, or delayed intervention effects. We additionally highlight the resulting uncertainty surrounding long-term ICER estimates and interpretation of cost-effectiveness findings.

Comment 4

Long-term evidence for emerging pharmacological therapies remains limited.

Response

We added discussion acknowledging that robust long-term economic evaluations of emerging pharmacological weight-loss therapies will require evidence regarding durability of treatment effects, long-term adherence, adverse events, and sustainability of weight loss. We note that such evidence is currently limited and is likely to emerge over the medium to long term.

VERSION 2 - REVIEW

Reviewer 2

Name Herrmann, Stephan

Affiliation University of Freiburg Medical Center Freiburg, Department of General and Visceral Surgery

Date 06-Jul-2026

COI

Thank you for the revised manuscript.

One minor issues:

In the abstract section the number of identified references should be corrected from 1,410 to 1,014 to reflect the actual number after removal of duplicates.

VERSION 2 - AUTHOR RESPONSE

Reviewer's Comments

In the abstract section the number of identified references should be corrected from 1,410 to 1,014 to reflect the actual number after removal of duplicates.

The abstract has been corrected to report the accurate number of records identified after duplicate removal, changing the value from 1,410 to 1,014.
