# Supplementary material for: Systematic review of cost-effectiveness analyses of weight loss interventions for knee osteoarthritis
Source: BMJ Open. 2026 Sep 22;16(9):e117500. doi: 10.1136/bmjopen-2026-117500 (PMC13599885; doi:10.1136/bmjopen-2026-117500)
Supplement: Supplementary file 2 [file bmjopen-16-9-s001.docx]

***Supplementary Table 1: Study characteristics.***

| **Study** | | **Study Design/Type of economic evaluation** | | **Computer modelling used** | | **Country** | | **Population** | **Intervention** | | **Control** | **Main outcome measured** | | **Discount rate** | | **Time horizon** |
| --- | --- | --- | --- | --- | --- | --- | --- | --- | --- | --- | --- | --- | --- | --- | --- | --- |
| McLawhorn et al. 2016 | | CMB  CUA | | Markov Model based on observed population | | US | | BMI of >35 kg/m^2^ with end-stage knee osteoarthritis | Non specified bariatric surgery 2 years prior to TKR | | TKR | ICER, QALE | | 3% | | sim 40years |
| Kopp et al.2024 | | CMB  CUA | | OAPol Model based on observed population | | US | | Observed population based on WE CAN trial: People with knee OA and BMI >27.  The mean age ± SD was 65 ± 8 years, 77% were female, and mean ± SD BMI was 37 ± 7. The mean ± SD starting pain score was 38 ± 16 on the WOMAC scale | 1) health education program derived from the ADAPT trial.  Participants attended monthly 1-hour group sessions during the first 3 months, featuring educational presentations on osteoarthritis, obesity, and exercise. They were encouraged to follow American College of Rheumatology (ACR) and the European League Against Rheumatism (EULAR) recommendations for weight loss and physical activity, with question-and-answer sessions after each presentation. Follow-up contact occurred monthly during months 4–6 and every other month during months 7–18 to collect information on pain, medication use, illness, and hospitalization.  2) Diet and exercise program derived from the IDEA trial  Diet programme – Participants used up to 2 meal-replacement shakes daily, with calorie intake adjusted based on weekly weight changes. Diets created an 800–1000 kcal/day deficit, with minimum intakes of 1100 kcal/day for women and 1200 kcal/day for men. Meals emphasized 15–20% protein, <30% fat, and 45–60% carbohydrates, including a low-fat meal, healthy snacks, and individualized meal plans.  Exercise programme - 60-minute sessions, 3 times/week for 18 months. Sessions included 30 minutes of aerobic exercise, 20 minutes of strength training, and 10 minutes of cool-down. Walking was the primary aerobic activity, with cycling as needed, performed at 50–75% heart-rate reserve. Strength training targeted major lower-body and upper-body muscle groups | | Current nonoperative therapies (eg, NSAIDs, other pain medications, exercise therapy, corticosteroid injections) + TKR if needed | ICER, QALE, WOMAC, BMI | | 3% | | sim lifetime from 18months observed |
| Losina et al.2019 | | CMB CUA | | OAPol Model based on observed population | | US | | Observed population based on IDEA Trial: People with knee OA and BMI ≥30 The mean ± SD age of the patients was 66 ± 6 years,  72% were female, and the average BMI was 33.6. The mean ± SD baseline WOMAC pain score was 32.5 ± 15.5 (0–100 scale, with 100 = worst). | Intensive Diet and exercise programme derived from the IDEA trial resulting in a mean 10.6-kg  weight reduction:  Exact programme is the same as above.  . | | pharmacologic NSAIDs regimen, followed by TKA among those eligible and willing to undergo surgery, and revision TKA among those who failed primary TKA | ICER, QALE, BMI, WOMAC | | 3% | | sim lifetime from 24months observed |
| Kostic et al. 2023 March | | CMB CUA | | OAPol Model based on observed population | | US | | Observed population based on Bariatric Surgery (LABS) study Baseline characteristics included a mean ± SD age of 45 ± 4.25 years and BMI of 46.6 ± 2.38 | 1) RYGB 2) LSG | | Normal TKR without bariatric surgery | ICER, QALE, oxycodone use %, TKR % | | 3% | | sim lifetime observed 7 year |
| Kostic et al. 2023 Aug | | CMB CUA | | OAPol Model based on observed population | | US | | Observed population, patients with advanced knee OA and Class 3 obesity: Initial age of mean ± SD 58.8 ± 4.5 years and average BMI stratified by sex and race (42.9 to 46.5)  Patients started with a mean ± SD pain score of 54 ± 15 on the WOMAC pain scale, with a minimum pain of 40 | 1)RYGB 2) LSG 3) lifestyle nonsurgical weight loss (diet, exercise, weight loss medication) 4) Total knee replacement | | no TKR, no weight loss measures | ICER, QALE | | 3% | | sim lifetime |
| Betensky et al.2025 | | CMB CUA | | OAPol Model based on observed population | | US | | Observed population based on STEP trial  81.6% women and 88.9% White non-Hispanic, with a mean age of 56 years (SD, 10.0) and a mean BMI of 40.3 (SD, 7.2). The mean starting pain was 70.9 on the Western Ontario and McMaster Universities Osteoarthritis Index | 1) Diet and Exercise programme derived from the IDEA trial as described above. 2) semaglutide 3) tirzepatide 4) LSG 5)RYGB | | Normal treatment of osteoarthritis without any interventions | ICER, QALY, Median cost | | 0-5% | | sim lifetime |
| Harris et al.2023 | | RCT CUA | | N/A | | Australia | | 415 people with knee OA and overweight or obesity (BMI 28–40 ) who were members of an Australian private health insurance fund (Medibank) | 1) The 6-month exercise program included six videoconference sessions with a physical therapist, who provided a personalized home exercise and physical activity plan, self-management education, behavioural counselling, exercise equipment, printed materials, and website support.  2) The 6-month diet-plus-exercise program included the exercise intervention plus six videoconference sessions with a dietitian aimed at achieving ≥10% weight loss through a ketogenic very-low-calorie diet (two meal replacements and one low-carbohydrate meal daily, followed by transition to a healthy eating plan), supported by educational resources and the website. | | Given information about OA, treatment options, exercise/ physical activity, weight loss, managing pain, sleep, and “success stories” links to external websites for further information. | ICER, Quality of life (AQoL-8D) | | N/A | | 12months |
| o'brien et al.2018 | RCT CUA/CEA | | N/A | | Australia | | 129 Patients on a waiting list for an outpatient orthopaedic consultation for their knee osteoarthritis at the John Hunter Hospital in NSW, Australia | | | Participants received brief telephone education on weight loss and physical activity for knee osteoarthritis, then were referred to the NSW Get Healthy Information and Coaching Service, a government program offering 10 tailored coaching calls over 6 months focused on diet, exercise, and healthy weight. | usual care pathway on the waiting list to have an orthopaedic consultation and could progress to consultation if scheduled or surgery if recommended by the orthopaedic department. | | ICUR, BMI, knee pain intensity (11point NRS), WOMAC score | | N/A | 12months |

*TKR = Total Knee Replacement. RYGB = Roux-en-Y gastric bypass, LSG = laparoscopic sleeve gastrectomy*

*RCT = Randomised Controlled Trial, CMB = Computer based modelling*

*ICER = Incremental Cost-Effectiveness Ratio, QALY = Quality-Adjusted Life Year, QALE =Quality-Adjusted Life Year over lifetime. AQoL-8D = Assessment of Quality-of-Life scoring instrument, ICUR = Incremental Cost Utility Ratio, CUA = Cost utility analysis, CEA = Cost effectiveness analysis, WOMAC score – Western Ontario and McMaster Universities Osteoarthritis Index.*

*N/A = not applicable*

*Sim = simulated*

*BMI = Body Mass index,*

**Supplementary Table 2: Cost and benefit of interventions assessed**

| **Author** | **Perspective** | **Weight loss intervention** | **Direct cost of intervention standardised to 2024 GBP (rounded to the nearest £)** | **Indirect cost of intervention standardised to 2024 GBP (rounded to the nearest £)** | **Benefit assessed to derive QALY gains.** |
| --- | --- | --- | --- | --- | --- |
| McLawhorn et al. 2016 | Societal | Bariatric surgery | Total: £23,113 Range: £ 18,762 – £31,188 | N.R | Utility gain from bariatric surgery: +0.145  Higher utility in non-obese vs obese states Reduced revision and complication transitions |
| Kopp et al 2024 | Healthcare | Health education | Total: £1,390 (18 months) • Personnel: £1,204 (staff time for group education delivery) • Facility/classroom costs: £185 | N.R | BMI reduction: 1.4 kg/m²; pain reduction: 10.3 WOMAC points; discontinuation: 23%; utility gains from reduced pain + modest weight loss; benefits decay over up to 5 years; downstream effects on OA progression, TKA and revision risk |
|  | Societal |  |  | OA-related productivity loss: up to £1,021/year; TKA productivity loss:£3,026 (primary), £3,168 (revision) |  |
|  | Healthcare | Diet and exercise | Total:£4,790 (18 months) • Personnel: £3,540 (dietitians, intervention staff, exercise supervision) • Meal replacements: £705 (structured dietary substitution component) • Facility/gym costs: £545 (exercise classes, equipment access) | N.R | BMI reduction: 3.2 kg/m²; pain reduction: 12.6 WOMAC points; discontinuation: 21%; adverse events in 7.25% (QoL penalty 0.9832 for 1 year + $76 cost/event); benefits persist up to 5 years then taper; reduced TKA, revision TKA, and chronic OA states |
|  | Societal |  |  | OA-related productivity loss: up to £1,021/year; TKA productivity loss:£3,026 (primary), £3,168 (revision) |  |
| Losina et al 2019 | healthcare | Diet and exercise | Total: £1,151–£1,948 per participant over intervention period≈ £290(personnel) + £402 (meal replacements) + £531 (gym) per year for up to 2 years → ; plus modelled lifetime incremental cost increase vs UC (~£1,633) | N.R | BMI reduction ~2.3–2.8 kg/m²; WOMAC pain reduction ~4.9–6.8 points over 18 months; ~38% achieving little/no pain vs 22% exercise control. |
|  | societal |  |  | OA-related productivity loss (£918/year), caregiving costs (£999/year), and productivity losses from TKA (£2,931–£ 3,180 in surgery year); applied only when pain exceeds threshold (>40 WOMAC score) |  |
| Kostic et al 2023 March | healthcare | Roux-en-Y Gastric Bypass | Total: £22,374= £18,737 surgery + £1,615 surgeon fees + £1,111 anaesthesiologist fees + 7 visits (£130 each) + £211/year long-term follow-up | N.R | Knee pain reduction: ~31% initial reduction; Type 2 diabetes remission: 70% (baseline diabetics); Diabetes incidence RR: 0.163; BMI reduction: 23–47% weight loss (max over 2 years); utility loss from BMI ≥40 vs 35–40: −0.0375 QoL |
|  |  | Laparoscopic Sleeve Gastrectomy | Total: £19,500= £16,694 surgery + £1, 004surgeon fees + £889 anaesthesiologist fees + 7 visits (£130 each) + £211 /year long-term follow-up | N.R | knee pain reduction: assumed same as RYGB (~31% initial reduction); Type 2 diabetes remission: 51%; BMI reduction: 18–38% weight loss (max over 1 year) |
| Harris et al 2023 | healthcare | telehealth exercise | Total ≈£321 (6 months program cost)± £98 Breakdown: • Physio consultations: ~6 sessions (≈ £64 initial + £53 follow-ups) • Fitbit: £36 • Exercise bands: £4 • Booklets: £14 • Postage: £5 | • Inpatient: £987 ± £2,758 • Medical services: ~£119 • Pharmaceuticals: ~£138 • Diagnostics: ~£81 • Other health services: ~£75 | QoL-8D utility (0–1 scale) • Baseline: ~0.69 • 6 months: ~0.76 • 12 months: ~0.75 ➡ QALY gain vs control: +0.03 (0.01–0.05) Method: trapezoidal integration of AQoL-8D at baseline, 6, 12 months |
|  |  | telehealth diet and exercise | Total ≈ £1,298 (6 months program cost) Breakdown: • Physio sessions: same as exercise (£321 component included) • Dietitian consultations: 6 sessions (≈ £*85* initial +£49 follow-ups) • Meal replacements: £718 per participant • Portion plate: £2 • Fitbit: £36 • Booklets/materials: £37 | 1197 • Inpatient: £*670* ± £2,320 • Medical services: ~£103 • Pharmaceuticals: ~ £114 • Diagnostics: ~ £76 • Other services: ~ £79 | AQoL-8D utility (0–1 scale) • Baseline: ~0.70 • 6 months: ~0.79 • 12 months: ~0.78 ➡ QALY gain vs control: +0.05 (0.03–0.07) ➡ Increment vs exercise: +0.02 |
| O'Brien et al 2018 | healthcare | telehealth advice | £383 ( 10 individually tailored coaching calls, centred on national dietary and physical activity guidelines over 6 month) | N.R | Exact scores not recorded |
|  | societal |  |  | Total incremental societal cost: £738 (95% CI -£1780 to £3,764) (includes healthcare + medication –£19.73 + absenteeism +£77) | Exact scores not recorded |
| Betensky et al 2025 | Healthcare | Exercise and diet | £3,539 (unknown cost) | N.R | ~8% BMI reduction (year 1); ~49% knee pain reduction; improves utility via lower BMI class, reduced OA pain, modest MACE reduction (0%), no diabetes pharmacologic remission effect |
|  |  | Semaglutide | £6,177 (Wegovy, Novo Nordisk)/ £2,984 (Ozempic, Novo Nordisk) | N.R | ~15% BMI loss; ~57% knee pain reduction; ~68% diabetes remission; ~20% MACE risk reduction; improved utility via weight loss, pain reduction, reduced comorbidity burden |
|  |  | Tirzepatide | £7,287 (Zepbound, Eli Lilly)/ £2,984 (Mounjaro, Eli Lilly) | N.R | ~25–34% BMI loss (higher than semaglutide); ~58% knee pain reduction; ~79% diabetes remission; ~20% MACE risk reduction; largest utility gain among medications |
|  |  | Laparoscopic Sleeve Gastrectomy | £18,183( surgical cost)+ £208 annually for follow ups | N.R | Sustained BMI reduction (~23% year 1, maintained partially long-term); ~54% pain reduction; durable improvement in utility due to long-term weight maintenance |
|  |  | Roux-en-Y Gastric Bypass | £20,959 (surgical cost) + £208 annually for follow ups | N.R | Largest sustained weight loss (~34% year 1 with long-term maintenance); ~54% pain reduction; strongest long-term utility gain due to durable weight loss, metabolic benefits, and survival improvement |
| Kostic et al 2023 Aug | Healthcare | Roux-en-Y Gastric Bypass (AT 47BMI) | Total: £22,374= £18,737 surgery + £1,615 surgeon fees + £1,111 anaesthesiologist fees + 7 visits (£130 each) + £211/year long-term follow-up | N.R | ~23–47% weight loss, 70% diabetes remission, 31% knee pain reduction, reduced obesity disutility (BMI ≥40: −0.0375 QoL), delayed/reduced TKR, complication risks, mortality effects |
|  |  | Roux-en-Y Gastric Bypass ( AT 42BMI) |  | N.R |  |
|  |  | Laparoscopic Sleeve Gastrectomy ( At 47BMI) | Total: £19,500 = £16,694 surgery + £1, 004surgeon fees + £889 anaesthesiologist fees + 7 visits (£130 each) + £211 /year long-term follow-up | N.R | 18–38% weight loss, 51% diabetes remission, 31% knee pain reduction (assumed same as RYGB), reduced obesity disutility, delayed/reduced TKR, complications |
|  |  | Laparoscopic Sleeve Gastrectomy (At 42BMI) |  | N.R |  |
|  |  | lifestyle nonsurgical weight loss (At 47 BMI) | Year 1: £2,595; Years 2–4: £1531/year; orlistat cost: £4,576/year | N.R | 5–15% weight loss, 8.8% diabetes remission, reduced obesity disutility; NO sustained knee pain improvement assumed, and may increase TKR utilization |

*N.R = Not recorded in the study.*

*WOMAC score – Western Ontario and McMaster Universities Osteoarthritis Index.*

*AQoL-8D = Assessment of Quality-of-Life scoring instrument*
