# Supplementary material for: Systematic review of cost-effectiveness analyses of weight loss interventions for knee osteoarthritis
Source: BMJ Open. 2026 Sep 22;16(9):e117500. doi: 10.1136/bmjopen-2026-117500 (PMC13599885; doi:10.1136/bmjopen-2026-117500)
Supplement: Supplementary file 3 [file bmjopen-16-9-s002.docx]

**Appendix 1: Literature searches for Knee OA review**

Overview

| ***Bibliographic databases and clinical trials registers*** | | |
| --- | --- | --- |
| ***Initial searches*** | | |
| **Database** | **Date searched** | **Number of records** |
| MEDLINE All (via Ovid) | 19/06/25 | 237 |
| Embase (via Ovid) | 19/06/25 | 699 |
| Science Citation Index (via Web of Science) | 19/06/25 | 455 |
| Global Index Medicus (via World Health Organization) | 19/06/25 | 2 |
| Clinicaltrials.gov | 19/06/25 | 17 |
| **Total number of records retrieved: 1,410**  **Duplicates removed (EndNote): 290**  **Final number for screening: 1,120** | | |

**19/06/2025**

**Ovid MEDLINE(R) <1946 to June Week 2 2025>**

1	exp Osteoarthritis, Knee/	31233

2	(knee adj2 osteoarthrit*).mp.	33643

3	(knee adj2 OA).mp.	8623

4	1 or 2 or 3	34391

5	exp Weight Loss/	53734

6	exp Obesity/	284311

7	exp Diet, Reducing/	11704

8	(weight adj2 loss).mp.	117361

9	(diet* adj2 reduc*).mp.	23380

10	(caloric adj2 restrict*).mp.	10828

11	(obes* or overweight).mp.	445823

12	("GLP-1 receptor agonist*" or "glucagon-like peptide-1 receptor agonist*" or GLP1RA or "GLP-1 agonist*" or "GLP1 agonist*" or exenatide or bydureon or liraglutide or victoza or saxenda or lixisenatide or lyxumia or dulaglutide or trulicity or semaglutide or ozempic or wegovy or efpeglenatide or ecnoglutide or danuglipron or tirzepatide or mounjaro).ti,ab,kf.	11853

13	(bariatric surg* or gastric bypass or sleeve gastrectomy).mp.	37534

14	(behavio?r* adj2 therap*).mp.	74037

15	(digital adj2 (intervention* or app* or tool* or support)).mp.	9808

16	(body weight management or weight control or calorie control or nutritional counsel*).mp.	8526

17	(mobile health or telemedicine or telehealth or motivational interviewing).mp.	67760

18	("weight loss surgery" or "obesity surgery" or "gastric bypass" or "Roux-en-Y" or RYGB or "sleeve gastrectomy" or "vertical sleeve" or "biliopancreatic diversion" or "duodenal switch" or "adjustable gastric banding" or "laparoscopic banding").ti,ab,kf.	26118

19	5 or 6 or 7 or 8 or 9 or 10 or 11 or 12 or 13 or 14 or 15 or 16 or 17 or 18	699023

20	exp "Costs and Cost Analysis"/	279425

21	(economic* or cost or costs or costly or costing or price or prices or expenditure or expenditures or expense or expenses or financial or finance or finances or financed).ti,kf.	256960

22	(economic* or cost or costs or costly or costing or price or prices or pricing or expenditure or expenditures or expense or expenses or financial or finance or finances or financed).ab. /freq=2	352344

23	(cost* adj2 (effective* or utilit* or benefit* or minimi* or analy* or outcome or outcomes)).ab,kf.	193709

24	(value adj2 (money or monetary)).ti,ab,kf.	2887

25	exp Models, Economic/	16853

26	economic model*.ab,kf.	3854

27	Markov Chains/	17120

28	markov.ti,ab,kf.	25028

29	Monte Carlo Method/	34240

30	monte carlo.ti,ab,kf.	40701

31	exp Decision Theory/	14344

32	(decision* adj2 (tree* or analy* or model*)).ti,ab,kf.	38103

33	Quality-Adjusted Life Years/	17921

34	(QALY or QALYS).mp.	14068

35	(utilit* adj2 (score* or value* or health)).mp.	7610

36	(EuroQol or Euro Qol or Euro-Qol or EQ 5D* or EQ-5D* or EQ5D*).mp.	16858

37	(health utilities index or health-utilities-index or health-utilities index or health utility index or HUI).mp.	2130

38	(SF 6D* or SF-6D* or SF6D* or SF-12* or SF 12* or SF 12* or short form health survey).mp.	13468

39	(short form 36* or SF36* or SF-36* or SF 36*).mp.	29747

40	(DALY or DALYs or disability adjusted life year* or disability-adjusted life year*).mp.	7085

41	(cost effective* or cost utilit* or cost benefit* or cost consequence*).mp.	213290

42	(ICER* or incremental cost-effectiveness ratio*).mp.	12159

43	(cost adj2 (evaluation* or analy* or study or studies or effective* or benefit* or utili*)).mp.	275297

44	(economic adj2 (evaluation* or analy* or study or studies)).ti,ab,kf.	26238

45	((markov or decision or economic) adj3 model*).mp.	44059

46	(trial-based adj2 (economic* or evaluation* or analy*)).ti,ab,kf.	267

47	(within adj2 trial adj2 (economic* or evaluation* or analy*)).ti,ab,kf.	218

48	20 or 21 or 22 or 23 or 24 or 25 or 26 or 27 or 28 or 29 or 30 or 31 or 32 or 33 or 34 or 35 or 36 or 37 or 38 or 39 or 40 or 41 or 42 or 43 or 44 or 45 or 46 or 47	825030

49	4 and 19 and 48	241

**19/06/2025**

**Embase <1974 to 2025 June 18>**

1	exp knee osteoarthritis/	55812

2	(knee adj2 osteoarthrit*).ti,ab,kf.	36218

3	(knee adj2 OA).ti,ab,kf.	20761

4	1 or 2 or 3	62493

5	exp weight reduction/	274897

6	exp obesity/	774722

7	exp reducing diet/	11723

8	(weight adj2 loss).ti,ab,kf.	215557

9	(diet* adj2 reduc*).ti,ab,kf.	18860

10	(caloric adj2 restrict*).ti,ab,kf.	9013

11	(obes* or overweight).ti,ab,kf.	703014

12	("GLP-1 receptor agonist*" or "glucagon-like peptide-1 receptor agonist*" or GLP1RA or "GLP-1 agonist*" or "GLP1 agonist*" or exenatide or bydureon or liraglutide or victoza or saxenda or lixisenatide or lyxumia or dulaglutide or trulicity or semaglutide or ozempic or wegovy or efpeglenatide or ecnoglutide or danuglipron or tirzepatide or mounjaro).ti,ab,kf.	28466

13	(bariatric surg* or gastric bypass or sleeve gastrectomy).ti,ab,kf.	74017

14	(behavio?r* adj2 therap*).ti,ab,kf.	59882

15	(digital adj2 (intervention* or app* or tool* or support)).ti,ab,kf.	19036

16	(body weight management or weight control or calorie control or nutritional counsel*).ti,ab,kf.	14556

17	(mobile health or telemedicine or telehealth or motivational interviewing).ti,ab,kf.	80598

18	("weight loss surgery" or "obesity surgery" or "gastric bypass" or "Roux-en-Y" or RYGB or "sleeve gastrectomy" or "vertical sleeve" or "biliopancreatic diversion" or "duodenal switch" or "adjustable gastric banding" or "laparoscopic banding").ti,ab,kf.	57884

19	5 or 6 or 7 or 8 or 9 or 10 or 11 or 12 or 13 or 14 or 15 or 16 or 17 or 18	1351979

20	exp health care cost/	373813

21	(economic* or cost or costs or costly or costing or price or prices or expenditure or expenditures or expense or expenses or financial or finance or finances or financed).ti,kf.	381101

22	(economic* or cost or costs or costly or costing or price or prices or pricing or expenditure or expenditures or expense or expenses or financial or finance or finances or financed).ab. /freq=2	630824

23	(cost* adj2 (effective* or utilit* or benefit* or minimi* or analy* or outcome or outcomes)).ab,kf.	349487

24	(value adj2 (money or monetary)).ti,ab,kf.	4638

25	exp economic model/	4991

26	economic model*.ab,kf.	7218

27	exp markov chain/	20316

28	markov.ti,ab,kf.	43310

29	exp monte carlo method/	60348

30	monte carlo.ti,ab,kf.	71195

31	exp decision theory/	1927

32	(decision* adj2 (tree* or analy* or model*)).ti,ab,kf.	66552

33	exp quality-adjusted life year/	40932

34	(QALY or QALYS or quality adjusted life year*).ti,ab,kf.	37613

35	(utilit* adj2 (score* or value* or health)).ti,ab,kf.	15416

36	(EuroQol or Euro Qol or Euro-Qol or EQ 5D* or EQ-5D* or EQ5D*).ti,ab,kf.	38303

37	(health utilities index or health-utilities-index or health-utilities index or health utility index or HUI).ti,ab,kf.	4147

38	(SF 6D* or SF-6D* or SF6D* or SF-12* or SF 12* or short form health survey).ti,ab,kf.	24133

39	(short form 36* or SF36* or SF-36* or SF 36*).ti,ab,kf.	58651

40	(DALY or DALYs or disability adjusted life year* or disability-adjusted life year*).ti,ab,kf.	11477

41	(cost effective* or cost utilit* or cost benefit* or cost consequence*).ti,ab,kf.	311046

42	(ICER* or incremental cost-effectiveness ratio*).ti,ab,kf.	25000

43	(cost adj2 (evaluation* or analy* or study or studies or effective* or benefit* or utili*)).ti,ab,kf.	353096

44	(economic adj2 (evaluation* or analy* or study or studies)).ti,ab,kf.	45168

45	((markov or decision or economic) adj3 model*).ti,ab,kf.	63499

46	(trial-based adj2 (economic* or evaluation* or analy*)).ti,ab,kf.	424

47	(within adj2 trial adj2 (economic* or evaluation* or analy*)).ti,ab,kf.	342

48	20 or 21 or 22 or 23 or 24 or 25 or 26 or 27 or 28 or 29 or 30 or 31 or 32 or 33 or 34 or 35 or 36 or 37 or 38 or 39 or 40 or 41 or 42 or 43 or 44 or 45 or 46 or 47	1393019

49	4 and 19 and 48	708

50	limit 49 to english language	699

**Web of Science 19/06/2025**

1	TS=(knee NEAR/2 osteoarthritis OR knee NEAR/2 OA)	46.499

2	TS=(("weight NEAR/2 loss") OR "reducing diet*" OR "caloric NEAR/2 restriction*" OR obes* OR overweight OR

"GLP-1 receptor agonist*" OR "glucagon-like peptide-1 receptor agonist*" OR GLP1RA OR

"GLP-1 agonist*" OR "GLP1 agonist*" OR exenatide OR bydureon OR liraglutide OR victoza OR saxenda OR

lixisenatide OR lyxumia OR dulaglutide OR trulicity OR semaglutide OR ozempic OR wegovy OR efpeglenatide OR

ecnoglutide OR danuglipron OR tirzepatide OR mounjaro OR

"bariatric surgery" OR "gastric bypass" OR "sleeve gastrectomy" OR "Roux-en-Y" OR RYGB OR

"vertical sleeve" OR "biliopancreatic diversion" OR "duodenal switch" OR

"adjustable gastric banding" OR "laparoscopic banding" OR

(behavio* NEAR/2 therap*) OR

(digital NEAR/2 (intervention* OR app* OR tool* OR support*)) OR

"body weight management" OR "weight control" OR "calorie control" OR "nutritional counsel*" OR

"mobile health" OR telemedicine OR telehealth OR "motivational interviewing")	901,084

3	TS=(economic* OR cost OR costs OR costly OR costing OR price OR prices OR pricing OR

pharmacoeconomic* OR expenditure OR expenditures OR expense OR expenses OR financial OR finance OR finances OR

financed OR "cost NEAR/2 (effectiveness OR utility OR benefit OR minimization OR analysis OR outcome OR outcomes)" OR

"value NEAR/2 (money OR monetary)" OR

"economic model*" OR

"markov model" OR "monte carlo" OR

"decision NEAR/2 (tree OR model OR analysis)" OR

"QALY" OR "QALYs" OR "quality adjusted life year*" OR

(utilit* NEAR/2 (score* OR value* OR health)) OR

"EuroQol" OR "EQ-5D" OR "EQ 5D" OR "EQ5D" OR

"health utilities index" OR HUI OR

"SF-6D" OR "SF6D" OR "SF-12" OR "short form health survey" OR

"SF-36" OR "short form 36" OR

"DALY" OR "DALYs" OR "disability adjusted life year*" OR

"ICER" OR "incremental cost-effectiveness ratio" OR

"cost NEAR/2 (evaluation OR analysis OR study OR studies OR utility)" OR

"economic NEAR/2 (evaluation OR analysis OR study OR studies)" OR

("trial-based" NEAR/2 (economic* OR evaluation* OR analysis*)) OR

("within trial" NEAR/2 (economic* OR evaluation* OR analysis*)))	5,347,858

4	#1 AND #2 AND #3 and English (Languages)	455

**Global Index Medicus (via World Health Organization) 19.06.2025**

("knee osteoarthritis" OR "knee OA")

AND

("weight loss" OR "reducing diet" OR "caloric restriction" OR obes* OR overweight OR

"GLP-1 receptor agonist*" OR "GLP1 agonist*" OR exenatide OR liraglutide OR saxenda OR semaglutide OR ozempic OR wegovy OR dulaglutide OR trulicity OR tirzepatide OR mounjaro OR

"bariatric surgery" OR "gastric bypass" OR "sleeve gastrectomy" OR "Roux-en-Y" OR RYGB OR

"vertical sleeve" OR "duodenal switch" OR "adjustable gastric banding" OR "laparoscopic banding" OR

"behavior therapy" OR "digital intervention" OR "nutritional counseling" OR "mobile health" OR telemedicine OR telehealth)

AND

(cost OR costs OR "economic evaluation" OR "cost effectiveness" OR QALY OR DALY OR ICER OR "healthcare cost" OR "financial burden" OR "economic model")	4

Limit to English Language	2

**Clinicaltrials.gov 19.06.2025**

"knee osteoarthritis" OR "knee OA"

AND

("weight loss" OR "diet" OR "obesity" OR overweight OR "caloric restriction" OR "GLP-1 receptor agonist" OR GLP1RA OR exenatide OR liraglutide OR semaglutide OR dulaglutide OR trulicity OR ozempic OR wegovy OR tirzepatide OR mounjaro OR "gastric bypass" OR "bariatric surgery" OR "sleeve gastrectomy" OR RYGB OR "behavioral therapy" OR "digital health" OR "mobile health" OR telehealth OR telemedicine)

AND

("cost effectiveness" OR "economic evaluation" OR "cost analysis" OR QALY OR DALY OR ICER OR "economic model" OR "financial impact" OR "healthcare cost")	17

**Appendix 2: ICER standardisation tables**

| Study | Perspective | Intervention | Total cost of intervention | Cost of intervention standardised GBP2024 | Total cost of  comparator | Cost of comparator standardised GBP2024 | Effect size (QALY diff) | ICER new |
| --- | --- | --- | --- | --- | --- | --- | --- | --- |
| McLawhorn et al. 2016 | Societal | Bariatric surgery | 84099.00 | 78728.15 | 60453.00 | 56592.27 | 1.7 | 13021.10588 |
| Kopp et al 2024 | Healthcare | Health education | 143927.00 | 118413.25 | 142789.00 | 117476.99 | 0.07 | 13375.14286 |
|  | Societal |  | 148139.00 | 121878.60 | 147102.00 | 121025.43 | 0.08 | 10664.625 |
|  | Healthcare | Diet and exercise | 147355.00 | 121233.58 | 142789.00 | 117476.99 | 0.12 | 31304.91667 |
|  | Societal |  | 151478.00 | 124625.70 | 147102.00 | 121025.43 | 0.13 | 27694.38462 |
| Losina et al 2019 | healthcare | Diet and exercise | 118100.00 | 104556.21 | 116200.00 | 102874.10 | 0.054 | 31150.18519 |
|  | societal |  | 132400.00 | 117216.27 | 130700.00 | 115711.23 | 0.054 | 27871.11111 |
| Kostic et al 2023 March | healthcare | Roux-en-Y Gastric Bypass | 278344.00 | 229002.33 | 271135.00 | 223071.26 | 1.36 | 4361.080882 |
|  |  | Laparoscopic Sleeve Gastrectomy | 279502.00 | 229955.06 | 271135.00 | 223071.26 | 1.14 | 6038.421053 |
| Harris et al 2023 | healthcare | telehealth exercise | 1754.00 | 981.68 | 0.00 | 0.00 | 0.03 | 32722.66667 |
|  |  | telehealth diet and exercise | 2196.00 | 1229.06 | 0.00 | 0.00 | 0.05 | 24581.2 |
| O'Brien et al 2018 | healthcare | telehealth advice |  |  |  |  | 0 | #DIV/0! |
|  | societal |  |  |  |  |  | 0 | #DIV/0! |
| Betensky et al 2025 | Healthcare | Exercise and diet | 232900.00 | 161632.6 | 229000.00 | 158926 | 0.16 | 16916.25 |
|  |  | Semaglutide | 276500.00 | 191891 | 229000.00 | 158926 | 0.92 | 35831.52174 |
|  |  | Tizepatide | 282000.00 | 195708.00 | 229000.00 | 158926 | 1.14 | 32264.91228 |
|  |  | Laparoscopic Sleeve Gastrectomy | 249800.00 | 173361.2 | 229000.00 | 158926 | 1.01 | 14292.27723 |
|  |  | Roux-en-Y Gastric Bypass | 257700.00 | 178843.80 | 229000.00 | 158926 | 1.27 | 15683.30709 |
| Kostic et al 2023 Aug | Healthcare | Roux-en-Y Gastric Bypass ( AT 47BMI) | 263216.00 | 216556.05 | 241262.00 | 198493.81 | 1.12 | 16127 |
|  |  | Roux-en-Y Gastric Bypass ( AT 42BMI) | 250805.00 | 206345.13 | 227578.00 | 187235.55 | 0.99 | 19302.60606 |
|  |  | Laparoscopic Sleeve Gastrectomy ( At 47BMI) | 258609.00 | 212765.73 | 241262.00 | 198493.81 | 0.89 | 16035.86517 |
|  |  | Laparoscopic Sleeve Gastrectomy (At 42BMI) | 244540.00 | 201190.72 | 227578.00 | 187235.55 | 0.81 | 17228.60494 |
|  |  | lifestyle nonsurgical weight loss ( At 47 BMI) | 258107.00 | 212352.72 | 241262.00 | 198493.81 | 0.15 | 92392.73333 |
|  |  | lifestyle nonsurgical weight lossR ( At 42 BMI) | 244350.00 | 201034.4 | 227578.00 | 187235.55 | 0.18 | 76660.27778 |

**Appendix 3: Methodological quality appraisal of included studies : Consolidated Health Economic Evaluation Reporting Standards 2022 (CHEERS 2022)**

| CHEERS checklist | | | | | | | | | | | | | | | | | | | | | | | | | | | | | Overall reported items | % of total | |
| --- | --- | --- | --- | --- | --- | --- | --- | --- | --- | --- | --- | --- | --- | --- | --- | --- | --- | --- | --- | --- | --- | --- | --- | --- | --- | --- | --- | --- | --- | --- | --- |
| Study | 1 | 2 | 3 | 4 | 5 | 6 | 7 | 8 | 9 | 10 | 11 | 12 | 13 | 14 | 15 | 16 | 17 | 18 | 19 | 20 | 21 | 22 | 23 | 24 | 25 | 26 | 27 | 28 |  |  |  |
| McLawhorn et al. | 1 | 1 | 1 | 1 | 1 | 1 | 1 | 1 | 1 | 1 | 1 | 1 | 1 | 1 | 1 | 1 | 1 | 1 | 0 | 1 | 0 | 1 | 0 | 1 | 0 | 1 | 1 | 1 | 24 | 86% |  |
| Kopp et al | 1 | 1 | 1 | 1 | 1 | 1 | 1 | 1 | 1 | 1 | 1 | 1 | 1 | 1 | 1 | 1 | 1 | 1 | 0 | 1 | 0 | 0 | 0 | 1 | 0 | 1 | 0 | 1 | 22 | 79% |  |
| Losina et al | 1 | 1 | 1 | 1 | 1 | 1 | 1 | 1 | 1 | 1 | 1 | 1 | 1 | 1 | 1 | 1 | 1 | 1 | 0 | 1 | 0 | 1 | 0 | 1 | 0 | 1 | 0 | 1 | 23 | 82% |  |
| Kostic et al Mar | 1 | 1 | 1 | 1 | 1 | 1 | 1 | 1 | 1 | 1 | 1 | 1 | 1 | 1 | 1 | 1 | 1 | 1 | 0 | 1 | 0 | 1 | 0 | 1 | 0 | 1 | 0 | 1 | 23 | 82% |  |
| Kostic et al Aug | 1 | 1 | 1 | 1 | 1 | 1 | 1 | 1 | 1 | 1 | 1 | 1 | 1 | 1 | 1 | 1 | 1 | 1 | 0 | 1 | 0 | 1 | 0 | 1 | 0 | 1 | 0 | 1 | 23 | 82% |  |
| Betensky et al | 1 | 1 | 1 | 1 | 1 | 1 | 1 | 1 | 1 | 1 | 1 | 1 | 1 | 1 | 1 | 1 | 1 | 1 | 0 | 1 | 0 | 1 | 0 | 1 | 0 | 1 | 1 | 1 | 24 | 86% |  |
| Harris et al | 1 | 1 | 1 | 1 | 1 | 1 | 1 | 1 | 1 | 0 | 1 | 1 | 1 | 1 | 1 | 1 | 1 | 0 | 0 | 1 | 0 | 0 | 1 | 1 | 0 | 1 | 1 | 1 | 22 | 79% |  |
| o'brien et al | 1 | 1 | 1 | 1 | 1 | 1 | 1 | 1 | 1 | 1 | 1 | 1 | 1 | 1 | 1 | 1 | 1 | 0 | 0 | 1 | 0 | 0 | 1 | 1 | 0 | 1 | 1 | 1 | 23 | 82% |  |

**Appendix 4: Methodological quality appraisal of included studies: Drummond Checklist for Economic evaluations**

| **Domain** | **Question** | **McLawhorn et al.** | **Kopp et al.** | **Losina et al.** | **Kostic et al. Mar** | **Kostic et al. Aug** | **Betensky et al.** | **Harris et al.** | **O’Brien et al.** |
| --- | --- | --- | --- | --- | --- | --- | --- | --- | --- |
| 1 | Well-defined question posed? | Yes | Yes | Yes | Yes | Yes | Yes | Yes | Yes |
| 1.1 | Costs and effects included? | Yes | Yes | Yes | Yes | Yes | Yes | Yes | Yes |
| 1.2 | Comparison of alternatives? | Yes | Yes | Yes | Yes | Yes | Yes | Yes | Yes |
| 1.3 | Perspective stated? | Yes | Yes | Yes | Yes | Yes | Yes | Yes | Yes |
| 2 | Alternatives described? | Yes | Yes | Yes | Yes | Yes | Yes | Yes | Yes |
| 2.1 | Important alternatives omitted? | No | No | No | No | No | No | No | No |
| 2.2 | Do-nothing included? | No | Yes | Yes | Yes | Yes | Yes | Yes | No |
| 3 | Effectiveness established? | Yes | Yes | Yes | Yes | Yes | Yes | Yes | Yes |
| 3.1 | RCT used? | No | Yes | Yes | Yes | No | No | Yes | Yes |
| 3.2 | Systematic review used? | Partial | No | No | No | Partial | No | No | No |
| 3.3 | Observational/assumptions used? | Yes | Yes | Yes | Yes | Yes | Yes | Yes | Yes |
| 4 | Costs & consequences identified? | Yes | Yes | Yes | Yes | Yes | Yes | Yes | Yes |
| 4.1 | Range sufficient? | Yes | Yes | Yes | Yes | Yes | Yes | Partial | Yes |
| 4.2 | Viewpoints covered? | Yes | Yes | Yes | Yes | Yes | Yes | No | Yes |
| 4.3 | Capital & operating costs included? | Yes | Yes | Yes | Yes | Yes | Yes | Yes | Yes |
| 5 | Measured in appropriate units? | Yes | Yes | Yes | Yes | Yes | Yes | Yes | Yes |
| 5.1 | Omissions in measurement? | Yes | Yes | Yes | Yes | Yes | Yes | Yes | Yes |
| 5.2 | Special circumstances handled? | Yes | Yes | Yes | Yes | Yes | Yes | Yes | Yes |
| 6 | Costs & consequences valued credibly? | Yes | Yes | Yes | Yes | Yes | Yes | Yes | Yes |
| 6.1 | Sources clearly identified? | Yes | Yes | Yes | Yes | Yes | Yes | Yes | Yes |
| 6.2 | Market values used? | Yes | Yes | Yes | Yes | Yes | Yes | Yes | Yes |
| 6.3 | Adjustments where needed? | Yes | Yes | Yes | Yes | Yes | Yes | Yes | Yes |
| 6.4 | Appropriate valuation method? | Yes | Yes | Yes | Yes | Yes | Yes | Yes | Yes |
| 7 | Timing adjustments applied? | Yes | Yes | Yes | Yes | Yes | Yes | Yes | Yes |
| 7.1 | Costs discounted? | Yes | Yes | Yes | Yes | Yes | Yes | N/A | N/A |
| 7.2 | Discounting justified? | Yes | Yes | Yes | Yes | Yes | Yes | N/A | N/A |
| 8 | Incremental analysis performed? | Yes | Yes | Yes | Yes | Yes | Yes | Yes | Yes |
| 8.1 | Costs vs outcomes compared? | Yes | Yes | Yes | Yes | Yes | Yes | Yes | Yes |
| 9 | Uncertainty addressed? | Yes | Yes | Yes | Yes | Yes | Yes | Yes | Yes |
| 9.1 | Appropriate statistical analysis? | Yes | Yes | Yes | Yes | Yes | Yes | Yes | Yes |
| 9.2 | Sensitivity ranges justified? | Yes | Yes | Yes | Yes | Yes | Yes | Yes | Yes |
| 9.3 | Results sensitive? | Yes | Yes | Yes | Yes | Yes | Yes | Yes | Yes |
| 10 | All issues discussed? | Yes | Yes | Yes | Yes | Yes | Yes | Yes | Yes |
| 10.1 | ICERs interpreted correctly? | Yes | Yes | Yes | Yes | Yes | Yes | Yes | Yes |
| 10.2 | Compared with other studies? | Yes | Yes | Yes | Yes | Yes | Yes | Yes | Yes |
| 10.3 | Generalisability discussed? | Yes | Yes | Yes | Yes | Yes | Yes | Yes | Yes |
| 10.4 | Ethical/distributional issues? | No | Partial | Partial | Partial | Partial | No | No | No |
| 10.5 | Implementation discussed? | Yes | Yes | Yes | Yes | Yes | Yes | Yes | Yes |
